# Predictors for Salivary Gland Cancer Recurrence at Two Tertiary Hospitals in Saudi Arabia

**DOI:** 10.7759/cureus.5288

**Published:** 2019-07-31

**Authors:** Thamer Alraddadi, Saleh Aldhahri, Jabir Alharbi, Moayyad Malas, Tahera Islam, Ahmad Altuwaijri, Khalid Al-Qahtani

**Affiliations:** 1 Otolaryngology, King Saud University, Riyadh, SAU; 2 Otolaryngology, Majmaah University, Riyadh, SAU; 3 Otolaryngology, King Khaled Hospital, King Abdulaziz Medical City, Jeddah, SAU; 4 Miscellaneous, College of Medicine and Research Center, King Saud University, Riyadh, SAU

**Keywords:** salivary gland cancer, recurrence, predictors, advanced stage

## Abstract

Background

Tumors of salivary glands are enlarged as a low-risk mass and mostly benign in nature. The treatment of salivary gland malignancy remains quite challenging because of its relative infrequency, unreliable biological manners, and risk of re-emergence. For effective treatment planning, prediction of factors associated with the recurrence of salivary gland malignancy is essential.

Aim

The objective of this study was to identify the factors associated with recurrence of salivary gland malignancy at two tertiary hospitals in Saudi Arabia.

Material and methods

A cross-sectional study was conducted at two tertiary hospitals in Saudi Arabia, where data of patients were recorded from the medical records of hospitals from 2012 to 2018. 63 patients who were diagnosed with salivary gland malignancy, whether originated from parotid, submandibular, sublingual, and minor salivary glands were included in the study. Descriptive statistics are shown in mean, frequency, and percentages, while comparative analysis was done using the Chi-square test, where *p *< 0.05 is considered significant.

Results

This study included 63 participants. The majority of malignant salivary gland cancers arise from the parotid gland (47.6%), and mucoepidermoid carcinoma was the commonest form of malignancy in 36.5% of the studied population. Out of 63 patients, 17 had a recurrence of cancer, and the initial advanced stage of the disease is apparently associated with the re-emergence of salivary gland cancer.

Conclusion

The initial advanced stage of cancer has a significant role in the recurrence of salivary gland malignancy. As salivary gland malignancy is rare and has unreliable behavior, establishing an adequate treatment plan prediction for recurrence is essential.

## Introduction

Tumors of salivary glands are enlarged as a low-risk mass, with most of them benign in nature and only a few malignant [1]. The cancers of salivary glands are usually infrequent, occurring in 0.6-1.4 per 100,000 and may originate from either major or minor salivary glands. It accounts for 0.5% of all cancers and 5% of cancers associated with head and neck [2-4]. There are approximately 40 histological types of salivary glands tumors, with some of them astonishingly atypical. The prevalence of salivary gland benign tumors is about 54% to 79% and the malignant tumors about 21-46% [5]. Usually, the clinical behavior of the salivary gland tumors depend upon histology, grading, and TNM staging and classified into low-grade, intermediate, and high-grade categories [6]. In previous studies, the degree of differentiation correlates significantly with the frequency of lymph node metastases, the degree of local invasion, which further affects the overall survival rate [7-8]. Unlike most of head and neck cancers that generally arise due to adverse habits of smoking and drinking, the etiologic factor behind salivary gland malignancy remains unclear [9]. Radiation exposure during the treatment for head and cancers, occupational exposure, previous history of cancers in other parts of body, Epstein-Barr Virus infection, immunosuppression, and HIV infections may be considered as menacing factors for salivary gland malignancy [10-13]. For that reason, diagnosis, the judgment of prognosis and choice of treatment in patients with salivary gland malignancies need to be enhanced [14].

In salivary gland tumors, multiple clinical and pathological variations present in major and minor salivary glands from different geographic areas. Studies from Iran and Sri Lanka reported a high incidence of malignant cancer in salivary glands [15-17] while the opposite has been reported from the USA and China [18-19]. Salivary gland cancers are relatively rare with unreliable behavior, considerable variations in racial and geographical distribution and risk of recurrence which makes the treatment quite challenging. The inadequate data are available in regards to the factors associated with the recurrence of salivary gland malignancy. Thus, the purpose of this study was to identify and highlights the factors associated with recurrence of salivary gland malignancy in two tertiary (national) hospitals in Saudi Arabia.

## Materials and methods

A cross-sectional study was conducted on all patients discussed in the tumor board with salivary gland tumors from 2012 to 2018 in two tertiary hospitals in Saudi Arabia. The centers included in this study are located in the central region in the Kingdom of Saudi Arabia and are considered the main referral centers for head and neck cancer patients from around the country, especially those with advanced disease and comorbidities. The medical records of the included patients were retrieved from the hospitals’ medical record department, and data of patients were recorded in a structured manner. Ethical approval for the study was obtained from the institutional ethical committee and administrative department of the hospitals. All patients diagnosed with salivary gland malignancy originating from the parotid, submandibular, sublingual, and minor glands were included in the study. Patients who lost follow-up or have incomplete data were excluded from this study.

Data collection included participants’ demographics and tumor data. Age, gender, stage of cancer, and geographic location of the patients were considered as credential factors for the possibility of recurrence of the salivary gland tumors. Also, disease characteristics, such as patients’ presenting symptoms, site of lesions, incision biopsy results, histopathology, and presence of recurrence were recorded. TNM staging was performed according to the Seventh Edition of the American Joint Committee on Cancer (AJCC), Cancer Staging Manual [20]. Statistical analysis was done using Statistical Package for the Social Sciences (SPSS) software, version 20. Descriptive statistics are shown as mean, frequency, and percentages, while comparative analysis was done using the Chi-square test. Statistical significance was considered when p-value <0.05.

## Results

Initially, 101 patients were recruited for the purpose of this study, but 38 were excluded who were not available for follow-up. The total sample size comprised 63 patients. Approximately half of the participants were females (55.6%). The mean age of the included sample was 44.8 years, and 39.6% of the patients were older than 50 years. The Kingdom of Saudi Arabia is divided into five regions. Geographic distribution of the patients showed that among the included participants, 37 were from the central region, 13 were from the south region, six were from the east and west regions, and only one came from the north region. The demographic characteristics of the study participants are shown in Table 1.

According to our data, lumps were the most frequently occurring symptom in 49 patients followed by pain and facial nerve paralysis. According to the site of lesions, the majority of lesions originated from the parotid gland (47.6%) followed by palate (20.6%), submandibular gland (11.1%), and 20.6% in other locations. In this study, an incisional biopsy was performed only in four patients. TNM staging of cancer showed that 27 patients were at stage IV, 11 were at stage III, 13 were at stage II, and 10 were at stage I. Histopathological examination of the tumors showed that the most frequent type of cancer is mucoepidermoid carcinoma presenting in 36.5% followed by adenoid cystic carcinoma (27%), salivary duct carcinoma (9.5%), acinic cell carcinoma, adenocarcinoma, epithelial-myoepithelial carcinoma (4.8%), carcinoma ex-pleomorphic adenoma (3.2%), squamous cell carcinoma (1.6%). Recurrence of salivary gland cancer was present in 17 patients. The data of disease characteristics have been shown in Table 2.

In the present study, old age, advanced stage of cancer, gender, and geographic location were considered as factors associated with the recurrence of salivary gland malignancy. Old age and gender were not associated with recurrence; however, the only factor associated with recurrence is the initial advanced stage of the tumor (*p *= 0.04), as shown in Table 3. As per the geographic location, the frequency of recurrence in the central region was highest followed by the south, east, west, and north regions.

**Table 1:** Demographics characteristics of study participants

Demographic characteristics	Frequency	Percentage (%)
Gender		
Male	28	44.4
Female	35	55.5
Region		
Central	37	58.7
East	6	9.5
West	6	9.5
South	13	20.6
North	1	1.6
Age >50 years	25	39.6

**Table 2:** Factors associated with recurrent disease

Factors	Frequency with recurrence	Frequency without recurrence	p-value
Old age (>50 years)	8	17	0.67
Advanced stage (III and IV)	14	24	0.044
Gender (male)	10	8	0.41
Geographic locations (Central-East-West-South-North)	8-2-2-5-0	29-4-4-8-1	

**Table 3:** Disease characteristics of study participants

Disease characteristic	Frequency	Percent
Presenting symptom		
Pain	5	7.9
Lump	49	77.8
Facial nerve paralysis	1	1.6
Other	8	12.7
Site of lesion		
Parotid	30	47.6
Submandibular	7	11.1
Palate	13	20.6
Other	13	20.6
Incisional biopsy performed	4	6.5
Stage		
0	1	1.6
I	10	16.1
II	13	21
III	11	17.7
IV	27	43.5
Histopathology		
Mucoepidermoid	23	36.5
Adenoid cystic	17	27
Acinic cell	3	4.8
Carcinoma ex-pleomorphic adenoma	2	3.2
Squamous cell Ca	1	1.6
Adenocarcinoma	3	4.8
Epithelial-myoepithelial carcinoma	3	4.8
Salivary duct carcinoma	6	9.5
Other	5	7.9
Presence of recurrence	17	27

## Discussion

In this cross-sectional study, we attempted to identify the factors associated with the recurrence of salivary gland malignancy. Salivary gland malignancy is uncommon and having changeable behavior which makes the treatment difficult. Therefore, prediction of factors associated with recurrence makes treatment planning easier and increases the overall survival rate of the patients. The demographic distribution of this case-control study showed slightly more female distribution with the mean age of 44.4 years. In contrast, previous studies conducted by Feinstein MT et al. and Sheddi MAA showed a male predominance [21-22].

According to this study, a lump was the most frequent symptom (77.8%) reported by patients. Similarly, Naami AL et al. found that a painless lump was a frequent manifestation observed in 71.8% of patients [23]. In the present study, the majority of malignancies originated from the parotid gland (47.6%), and similar results were found by Feinstein MT et al. and Eneroth CM [21,24]. In our study, mucoepidermoid carcinoma (36.5%) was the most common histologic variant followed by adenoid cystic carcinoma, salivary duct carcinoma, acinic cell, and adenocarcinoma. Similar results were replicated by Pinkston et al. [25]. On the contrary, a study by Feinstein MT et al. and Sheddi MAA identified adenoid cystic carcinoma as the most common histologic variant [21-22].

Our study found that the recurrence of salivary gland cancer is significantly associated with the TNM staging and higher recurrence rate was found in the initial advanced stages (III, IV) of cancer. Similar results were obtained by Jang JY et al. who presented that early-stage of disease (T1-2N0M0) showed apparently excellent prognosis and overall survival [26]. In contrast to previous studies, this study did not show a significant association between recurrence factors such as the age of patients, gender, and geographic location. Alkhateeb et al. reported that high predilection of mucoepidermoid carcinoma in minor salivary glands was associated with high female to male ratio, but recurrence does not depend upon gender [27]. Feinstein MT et al. showed that the age at the time of diagnosis had a significant relationship with overall survival (*p *= 0.01). However, age at diagnosis did not affect the risk of recurrence [21]. Due to the rarity and multiplicity of salivary gland cancers, the sample size of this study was small and hence, interpretation of the factors associated with the recurrence of salivary gland malignancies was quite challenging. Further study with larger sample size is recommended. The limitation of this study is a small sample size but this due to low incidence rate of the salivary glands cancer . 

## Conclusions

The initial advanced stage of cancer has a pivotal role in the recurrence of salivary gland malignancy. As salivary gland malignancy is rare with an unreliable biological behavior, its treatment remains challenging. Although our study did not show the association between recurrence and gender or old age, these factors were shown to be associated with the re-emergence of salivary gland malignancy in other studies, including the initial stage of cancer, age, gender, and geographic location. Therefore, for effective treatment planning, prediction of factors associated with the recurrence of salivary gland malignancy is mandatory.
